# What Are the Best Questionnaires To Capture Anorectal Function After Surgery in Rectal Cancer?

**DOI:** 10.1007/s11888-014-0217-6

**Published:** 2014-06-27

**Authors:** Tina Yen-Ting Chen, Katrine J. Emmertsen, Søren Laurberg

**Affiliations:** Department of Surgery P, Aarhus University Hospital, Tage-Hansens Gade 2, 8000 Aarhus C, Denmark

**Keywords:** Rectal cancer, Rectal cancer surgery, Anorectal function, Anorectal dysfunction, Bowel dysfunction, Low anterior resection syndrome, Anterior resection syndrome, Functional outcome, Sphincter-preserving surgery, Low anterior resection, Questionnaire, Scoring system, Instrument, Tool, Assessment, Measurement, Evaluation

## Abstract

With enhanced surgical techniques and neoadjuvant therapy in rectal cancer, survivorship issues are at the forefront of clinical practice and research. More and more patients are living with altered bowel habits following rectal cancer surgery. Sound assessment of anorectal function after rectal cancer surgery is the foundation for the continuing effort to explore the adverse effects of such surgery on bowel function, as well as for working towards reducing these effects. The quality of the assessment is predominantly determined by the instrument administered. This article reviews various questionnaires for capturing anorectal function after surgery in rectal cancer, discussing their attributes and suitability for different evaluation contexts.

## Introduction

Rectal cancer is common and poses much burden worldwide [1, 2]. Advances in treatment over the past three decades have led to substantially improved local control and survival [3–7]. Furthermore, the increasing use of sphincter-preserving resection with a low colorectal or coloanal anastomosis has resulted in fewer patients requiring a permanent colostomy, and it has become the standard surgery for mid and low rectal cancers [8–11].

Unfortunately, numerous patients experience anorectal, urinary, and/or sexual dysfunction after sphincter-preserving resection for rectal cancer [12•, 13•, 14–17], with anorectal dysfunction being the commonest, especially when the surgery is combined with radiotherapy [12•, 18–23].

## Low Anterior Resection Syndrome

Anorectal or bowel dysfunction after sphincter-preserving surgery manifests itself in a wide spectrum of symptoms, including fecal incontinence, increased stool frequency, urgency, and emptying difficulties. The complex of symptoms is often collectively referred to as anterior resection syndrome (ARS) or low anterior resection syndrome (LARS), named after the low anterior resection procedure [24]. Previously thought to be transient, LARS symptoms have been shown to persist for years after resection, suggesting that LARS is more of a permanent phenomenon [13•, 25]. These symptoms usually appear immediately after surgery, becoming most pronounced during the first few months, and improve somewhat thereafter, reaching a steady state after around one to two years [26].

Although LARS has been well recognized, it has not been clearly defined, with a large variation in the prevalence reported. LARS has been reported to affect up to 60–90 % of patients following low or ultralow anterior resection [13•, 24, 27–31], and can have a considerable impact on quality of life (QOL) [26, 30, 32–35]. Recently, a pragmatic definition of LARS has been proposed as “disordered bowel function after rectal resection, leading to a detriment in quality of life” [13•].

## Assessment of Anorectal Function After Rectal Cancer Surgery

Even though anorectal function, like other types of physical function, can be evaluated objectively to a certain extent, the patient’s own rating should be the gold standard, as only the patient can experience the function and perceive its true implications in the context of his or her life [36, 37]. A newly published study has demonstrated that even rectal cancer experts do not have a thorough understanding of which LARS symptoms truly matter to the patient, nor how these symptoms affect QOL, thus emphasizing the necessity of assessing anorectal function from the patient’s perspective [38].

## Nonvalidated or Unfitting Instruments

Many patient questionnaires or instruments have been used for assessing anorectal function after rectal cancer surgery. However, as highlighted in a systematic review and meta-analysis of long-term bowel function after curative anterior resection for rectal cancer, such assessment has been inconsistent due to the lack of a uniform definition of LARS, and the use of a large variety of nonvalidated questionnaires [12•]. It was found that 65 % of the studies included (48 studies in the qualitative analysis and 43 studies in the meta-analysis) did not use a validated assessment instrument [12•].

On the other hand, there are some anorectal function questionnaires that have been more rigorously tested, yet are not entirely suitable to be administered after surgery in rectal cancer; for instance, the American Medical Systems fecal incontinence scoring system [39], which was formulated for assessing the function of artificial anal sphincters, and the Bowel Function Questionnaire (BFQ) [40, 41], which focuses on symptoms during and after pelvic radiotherapy, as well as those caused by pharmacologic agents, instead of surgery [40–44].

A number of established bowel function-related QOL instruments, such as the European Organisation for Research and Treatment of Cancer Quality of Life Questionnaire Colorectal Module (EORTC QLQ-CR29) [45], the Fecal Incontinence Quality of Life Scale (FIQL) [46], and the Functional Assessment of Cancer Therapy – Colorectal (FACT-C) [47], have often been used to supplement the evaluation of anorectal function following surgery in rectal cancer, and to validate anorectal function questionnaires. This is because anorectal function questionnaires may only provide insight into symptoms, and not insight into how the patient is coping with the symptoms or how the patient’s life is disrupted by the symptoms. Although these various QOL instruments do contain some questions enquiring directly about anorectal function, they also contain questions on other matters. More importantly, although the fundamental constructs of function and QOL are related (function, as reflected in dysfunction symptoms, is a component and determinant of overall QOL), they are not the same [48, 49], and it would be conceptually flawed to use instruments designed for measuring the effects of bowel issues on QOL to specifically capture anorectal function.

There are questionnaires that would be more appropriate than those mentioned above for capturing anorectal function after surgery in rectal cancer. The rest of this article appraises these various validated and more fitting instruments.

## Fecal Incontinence Instruments

Fecal incontinence has long been the central focus of bowel dysfunction in general. In keeping with this, several well-known fecal incontinence questionnaires have been used in different studies to evaluate incontinence in LARS patients [50–57], including the Cleveland Clinic Florida (CCF)/Wexner Fecal Incontinence Score (Wexner score; 1993) [58], the St. Mark’s Incontinence Score (St. Mark’s score; 1999) [59], and the Fecal Incontinence Severity Index (FISI; 1999) [60].

The Wexner score is the most widely applied fecal incontinence instrument to date. It examines the frequency of three types of fecal incontinence (solid, liquid, and gas) and their consequences (pad wearing and lifestyle alteration). For each item, the five frequency options range from never (score 0) through to always (meaning at least once per day; score 4). The total score is the sum of the item scores, and ranges from 0 (perfect continence) to 20 (complete incontinence).

The St. Mark’s score is built on the Wexner score, incorporating three modifications [59]. Firstly, an urgency item (lack of ability to defer defecation for 15 min) was introduced, with dichotomous response options of no (score 0) and yes (score 4). Secondly, an item on antidiarrheal drugs (use of constipation medicines) was added, again with dichotomous response options of no (score 0) and yes (score 2). Lastly, it was thought that pad wearing should not be given the same emphasis as the incontinence items, because it is probably a measure of the patient’s degree of fastidiousness, instead of the severity of fecal incontinence [59]. Accordingly, the response options and scoring of the pad wearing item were adjusted to no (score 0) and yes (score 2). The total St. Mark’s score ranges from 0 (perfect continence) to 24 (complete incontinence).

The FISI investigates the frequency of four types of fecal incontinence (gas, mucus, liquid, and solid). There are six frequency options for every item, ranging from never through to two or more times per day. The score value of each incontinence type/frequency combination is derived from its severity ranking relative to other combinations, as rated by 34 patients and 26 colorectal surgeons. Therefore, two sets of scoring are available: a patient-based system and a surgeon-based system, with the patient-based system being more broadly used. The total score is the sum of the item scores, and the patient-based score ranges from 0 (no incontinence) to 61 (severe incontinence).

All three instruments were developed empirically, on the basis of known components of fecal incontinence [58–60]. They have been validated to varying extents, with the Wexner score being the least rigorously validated of the three, despite it being the most widely used. All three instruments have been proven to correlate with the patient’s subjective perception of bowel control [60–62]. They take on the same approach of assessing the frequency of incontinence episodes. This approach implies that frequency corresponds to severity, which may not always be the case. A minor symptom may occur often, but this does not mean that the symptom is severe. For example, a patient who experiences very slight leakage of liquid stool on a daily basis would score higher on these instruments compared with a patient who experiences leakage of a large amount of liquid stool every two days. The latter patient may not necessarily have better anorectal function, contrary to the impression given by these instruments.

Both the Wexner score and the St. Mark’s score use a linear scale for scoring the frequency of incontinence episodes, giving zero points to a symptom never occurring, one point to it occurring rarely (less than once per month), two points to it occurring sometimes (at least once per month but less than once per week), and so forth. This kind of scale assumes that the increase in anorectal dysfunction is linear throughout the response range. It also assumes that there is a linear correlation between the frequency a the symptom and its level of bother. However, evidence shows that there is a nonlinear relationship between symptom frequency and bother in patients with neurogenic bowel dysfunction [63]. In addition, in scoring the Wexner score and the St. Mark’s score, the different types of incontinence take on the same weighting, but the fact is that they (for example, incontinence for flatus vs. incontinence for solid stool) do not carry the same significance of anorectal dysfunction for either the patient or the clinician. Unlike the Wexner score and the St. Mark's score, the FISI uses patient- and surgeon-rated score values, and does not rely on a linear scale nor equal-weighting scoring.

The FISI uses a one month recall period, asking the patient to answer according to how often the symptom is experienced on average in the past month. In contrast, the Wexner score and the St. Mark's score do not specify a recall period. A recall period helps to evoke the patient's memory and ensures that the most up-to-date status is captured. Nonetheless, in some situations, a recall period may result in the response not being a fair representation of the patient's usual status, such as if the patient is currently having or has recently had an infection affecting bowel function.

 The most critical point to note about applying any fecal incontinence instrument to measure anorectal function after surgery in rectal cancer is that they only reveal the continence aspect. Because LARS is a disorder with heterogeneous symptoms and involves more than just incontinence, fecal incontinence instruments would not be able to fully capture the complexity of the problem.

## The Memorial Sloan-Kettering Cancer Center Bowel Function Instrument

The Memorial Sloan-Kettering Cancer Center Bowel Function Instrument (MSKCC BFI; 2005) is the first questionnaire designed specifically for evaluating bowel function after sphincter-preserving surgery for rectal cancer [64••]. It consists of 18 questions that enquire about the frequency of a variety of LARS issues. The MSKCC BFI adopts a four-week recall period, a linear scale, and equal-weighting scoring. For each question, the five frequency options range from never through to always (except for one question asking about the number of bowel movements per 24 hours). Response can be summarized into three subscales (frequency, six items; diet, four items; and urgency/soilage, four items) by adding the scores of the items in the subscale. The four single items that do not belong to a subscale look into incomplete evacuation, clustering (having another bowel movement within 15 min of the last movement), knowing the difference between needing to pass gas and a bowel movement, and incontinence for flatus. A global score can be calculated as the sum of the subscale scores, and a total score can be calculated by adding all the item scores (subscale plus single item scores). A higher score represents better bowel function. Some item scores require recoding before they can be used for computation.

The MSKCC BFI was meticulously formulated according to literature review, expert and patient input, factor analysis, and clinical relevance [64••]. Its psychometric precision, including the ability to detect differences in bowel function based on clinical variables, was confirmed through stringent validation [64••]. It has been translated from English into Italian, and this version has also been validated [65].

The main strengths of the MSKCC BFI are its scope and detail, allowing comprehensive and thorough evaluation of LARS. The subscales facilitate separate interpretation of different aspects of LARS when desired. However, the MSKCC BFI's length (18 questions) and scoring (which involves recoding, three subscale scores, a global score, and a total score) may influence its practicality.

## The Low Anterior Resection Syndrome Score

**Fig. 1:**
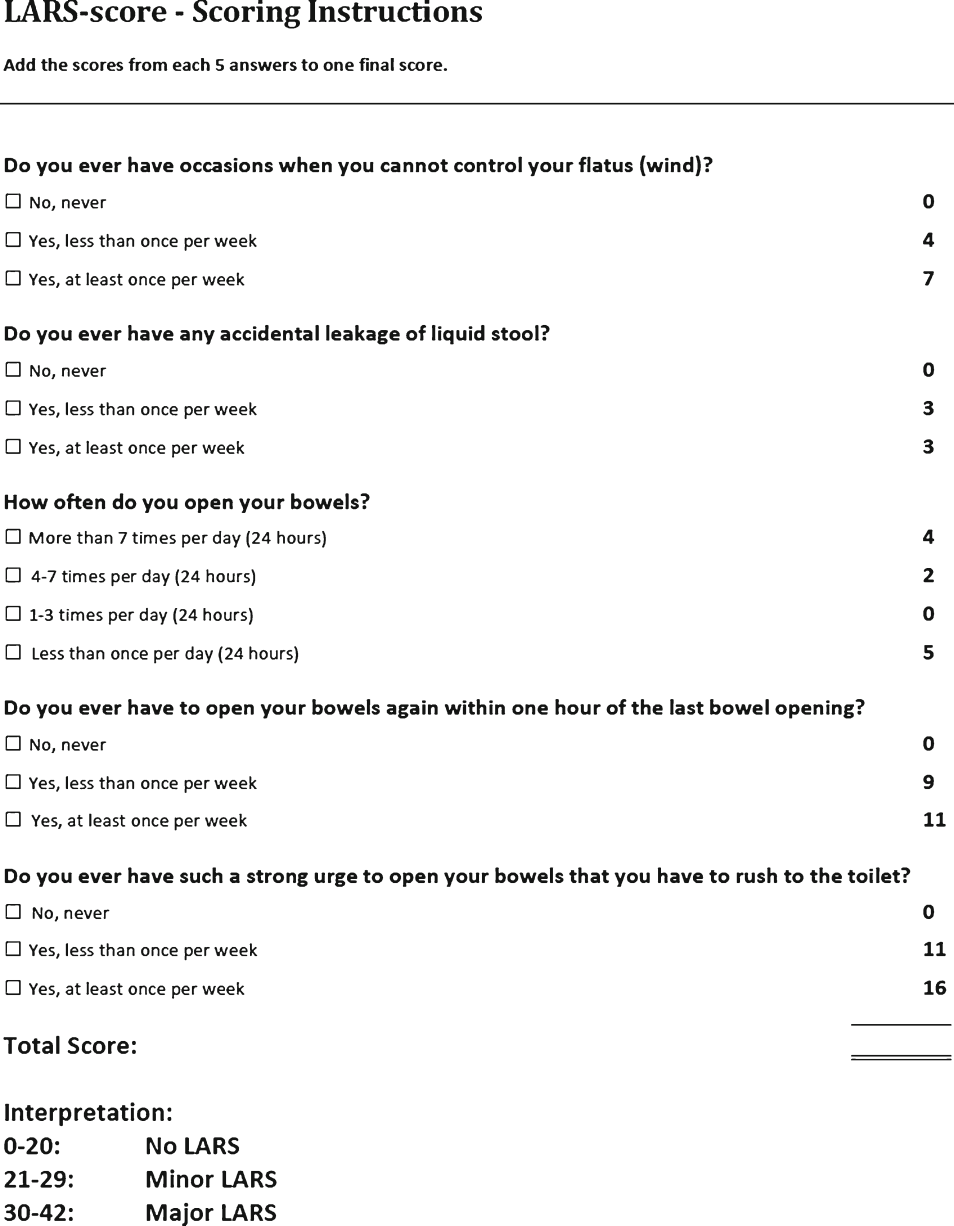
The Low Anterior Resection Syndrome Score (LARS score). (From Emmertsen and Laurberg [66••] with permission from Wolters Kluwer Health)

The Low Anterior Resection Syndrome Score (LARS score; 2012), like the MSKCC BFI, is a questionnaire for assessing bowel function after sphincter-preserving surgery with or without radiotherapy for rectal cancer [66••]. The items and scoring algorithm of the LARS score are shown in Fig. 1.

The LARS score was developed from and validated on a large, nationwide cohort of 961 Danish patients, who received curative low anterior resection with or without radiotherapy for nondisseminated rectal cancer in Denmark between 2001 and 2007 [66••]. By applying binomial regression on patient response, the five most bothersome LARS issues (both in terms of prevalence and impact on QOL), with at least one issue depicting each of the four known aspects of LARS (incontinence, frequency, urgency, and emptying difficulties), were selected for the LARS score from a pool of items extracted from existing bowel function questionnaires and the literature [66••]. The five issues selected are incontinence for flatus, incontinence for liquid stool, frequency (number of daily bowel movements), clustering (having to open bowels again within 1 h of the last opening), and urgency. The questions enquire about the frequency of symptom episodes. The LARS score does not use a specific recall period, a linear scale, nor equal-weighting scoring. The response score values, also derived using binomial regression, are based on the impact of the particular symptom/frequency combination on QOL [66••].

The ability of the LARS score to reflect the impact of bowel dysfunction on QOL was proven in its initial validation [66••], and subsequently through the association with many of the scales of the European Organisation for Research and Treatment of Cancer Quality of Life Questionnaire Core Module (EORTC QLQ-C30) [26, 67]. The impact on QOL is pertinent when assessing bowel dysfunction, because a mere description of the symptoms may not necessarily differentiate between patients with acceptable function and patients in need of further attention. In clinical settings, the LARS score severity categories (No, Minor, and Major LARS) can facilitate quick identification of patients most in need of treatment, namely those with Major LARS, since they also report seriously compromised QOL, and significantly worse QOL compared with those with No/Minor LARS [26, 66••, 67]. Moreover, the LARS score has demonstrated the ability to differentiate between subgroups of patients on the basis of clinical variables [66••]. In addition to the original Danish version, the LARS score has been translated into several other languages (English, Dutch, Swedish, Spanish, and German: validation is in progress for the former two, and the latter three have been validated in an international setting) [68], and hence has the capacity for widespread use.

The biggest strengths of the LARS score lie in its conciseness and ability to show impact on QOL. Its ease of scoring and clinically meaningful severity categories further support its routine use in clinical practice.

Urgency and clustering are the items with the highest response score values in the LARS score, indicating that these aspects of LARS affect the patient’s QOL the most. This reinforces the reasoning that fecal incontinence instruments and equal-weighting scoring would not be able to fully reflect LARS as experienced by the patient.

## Conclusions

Rectal cancer surgery is now performed with relatively good oncologic outcomes and the avoidance of a permanent colostomy in most cases. Nevertheless, many patients are plagued by LARS after such surgery. Sound assessment of anorectal function after rectal cancer surgery is the foundation for continuing to elucidate the true characteristics and incidence of LARS, for further improving surgical techniques to prevent LARS, and for trialing and consolidating the treatment of LARS. The quality of the assessment lies primarily in the questionnaire used.

Given that LARS is such a common problem that often leads to poor QOL, all patients should be routinely screened for LARS after sphincter-preserving surgery, and the level of anorectal function should be systematically recorded for benchmarking and quality improvement purposes. Consequently, routine and widespread assessment of LARS is called for.

In summary, the instruments appraised in this article have strengths and weaknesses that make up their own unique “attribute profile.” This profile determines the measurement context for which the instrument is most suitable.

For focused assessment of fecal incontinence, the Wexner score, the St. Mark’s score, and the FISI would all be adequate. Among the three instruments, the scoring and validation of the FISI are the most methodologically rigorous. It is vital that the application of fecal incontinence questionnaires is supported by proper rationale and accurate understanding of their limitations in painting the full picture of LARS.

For comprehensive and in-depth evaluation of LARS, the MSKCC BFI would be the questionnaire of choice. For rapid screening or assessment of LARS, the LARS score would be ideal. Both instruments are valid, reliable, and able to detect clinically relevant differences.

The use of one instrument does not preclude the use of another, and there may be situations where it is beneficial to use a combination of the aforementioned instruments.

All things considered, the MSKCC BFI and the LARS score are the best questionnaires to capture anorectal function after surgery in rectal cancer. Although there is some overlap in content, the two instruments are fundamentally different. Overall, the scope of the MSKCC BFI is broader, covering not only LARS symptoms, but also their consequences, such as diet limitations and pad wearing. On the other hand, the LARS score is more practical, and can indicate impact on QOL.

Finally, the adoption of a uniform definition of LARS, and the consistent use of the same questionnaires that best serve the particular measurement context and aim, are greatly encouraged, in order to pool and directly compare results of different studies, institutions, and interventions.
